# A Multiphase Composite for High-Performance Alkaline Zinc Batteries

**DOI:** 10.3390/molecules31111829

**Published:** 2026-05-26

**Authors:** Zhen Sun, Junran Wang, Jietao Guan, Yaoda Mei, Wenyu Song, Haixu Wang, Weiwei Luo, Xiang Cai

**Affiliations:** 1 Liaoning Key Laboratory of Development and Utilization for Natural Products Active Molecules, School of Chemistry and Life Science, Anshan Normal University, Anshan 114005, China; sunzhen@asnc.edu.cn (Z.S.); 13342351229@163.com (J.W.); 18841214631@163.com (J.G.); 13942011611@163.com (Y.M.); 15541058515@163.com (W.S.); wanghx918@nenu.edu.cn (H.W.); weiweiluo369@163.com (W.L.); 2 School of Light Industry and Chemical Engineering, Dalian Polytechnic University, Dalian 116034, China

**Keywords:** aqueous alkaline zinc battery, cathode material, multiphase composite

## Abstract

The development of high-performance cathode materials represents a crucial strategy for enhancing the overall electrochemical performance of aqueous alkaline zinc batteries. The rational design of electrode microstructure and chemical composition can synergistically boost the electrochemical reaction activity, ion/electron transport kinetics, and structural stability. In this work, a composite cathode material, FLG@Ni_x_S_6_/Co_4_S_3_/Ni-Co(OH)_2_, was successfully synthesized via an electrochemical codeposition method. The engineered architecture offers abundant electrochemically active sites, well-defined ion diffusion pathways, and continuous electron conduction networks. Moreover, the strong interaction among the constituent phases effectively regulates and accelerates the redox reaction kinetics. When integrated into an aqueous alkaline zinc battery, the device attains a high specific capacity of 385 mAh g^−1^ at 2 A g^−1^, excellent rate capability (287 mAh g^−1^ at 80 A g^−1^), a gravimetric energy density of 590 Wh kg^−1^, a power density of 128.57 kW kg^−1^, and remarkable cycling stability, with 100% capacity retention maintained after 20,000 cycles. Overall, this study proposes a scalable and rational composite strategy for designing high-performance electrode materials for next-generation electrochemical energy storage systems.

## 1. Introduction

Aqueous alkaline zinc batteries (AAZBs) have attracted widespread attention in the field of electrochemical energy storage owing to the use of zinc metal anodes which show the merit of a low electrode potential of −1.26 V versus SHE (Zn(OH)_4_^2−^ + 2e^−^ ↔ Zn + 4OH^−^), a high theoretical capacity of 820 mAh g^−1^, and relatively low cost [1,2,3]. However, the irreversible structural and constituent change of zinc metal anodes during repeated charge and discharge processes always gives rise to moderated cycling stability [4,5,6,7,8]. For example, the growth of Zn dendrites leads to cell shorting while the generation of the insulating ZnO from Zn(OH)_4_^2−^ ions in electrolytes easily passivates the surfaces of zinc metal anodes also resulting in rapid cell failure [9,10,11,12]. Fortunately, clear mechanistic pictures have gradually emerged thanks to considerable theoretical and experimental studies on the deposition–exfoliation behavior of zinc metal anodes in alkaline electrolytes [4,5,6]. The high solubility of discharge products and the spatially inhomogeneous kinetics of zinc deposition together lead to a loose zinc deposition morphology, unstable interfacial contact, and aggravated side-reactions [7,8]. To address these bottleneck issues, a variety of modification strategies have been comprehensively investigated, which primarily include the introduction of functional additives, anode surface engineering, and structural optimization design [9,10,11].

Although flourishing for studies on the failure behavior of zinc metal anodes, a matching cathode with a long-term lifespan is still scarce. In traditional AAZBs, Ni-based hydroxides with a hydrotalcite-like structure (α phase) were proposed as a class of feasible cathode materials [12]. They possess a high electrode potential and are characterized by multiple-electron transfer electrochemistry realized by the two-phase reaction from α to γ phases (γ-NiOOH_2−x_ + xH_2_O + xe^−^ ↔ α-Ni(OH)_2_ + xOH^−^, x > 1) [1]. However, γ-NiOOH_2−x_ is unstable in alkaline electrolytes and easily transforms into low-capacity β-NiOOH_2−x_ whose x value is normally below 1 [3,13,14]. Thus, hydrotalcite-like Ni-based hydroxides also have to face the challenges of an inferior lifespan. In other words, the mismatching between zinc metal anodes and Ni-based hydroxide cathodes in electrochemical performance necessitates the latter to be targetedly adjusted in their structure and composition [15,16,17,18]. It is a feasible strategy to enhance α-Ni(OH)_2_ by substituting a portion of nickel cations in the host layers with other transition metal cations such as Co^2+^ to synthesize Ni-based double hydroxides (DHs) [3,19,20,21]. Although the propose of DHs benefits effective suppression of the notorious two-phase reaction and a high Co^2+^ content can improve the electronic conductivity of α-Ni(OH)_2_ as well, the Co^2+^ centers with a lower faradic activity and an increase in the average valence state of the metal ion centers caused by the spontaneous insertion of anions into their interlayers inevitably promotes the loss of the theoretical capacity of α-Ni(OH)_2_ [17,22,23]. It is well known that the scientific fabrication of an anode and a cathode needs to take a multidimensional balance of not only stability but also capacity into consideration to minimize N/P ratios [24,25]. Namely, a high capacity is also indispensable for the advanced cathodes of AAZBs without compromising their lifespans to match their high-capacity zinc metal anodes.

Given this, in this work, a composite electrode was presented. The active materials (Ni_x_S_6_/Co_4_S_3_/Ni(OH)_2_·0.75H_2_O/Co(OH)_2_) were in situ grown on a cathode-exfoliated carbon electrode, with few-layer graphene (FLG) on top, via a facile electrochemical strategy. The multiple synergistic effects among the four components endow the composite electrode with not only a high specific capacity of 385 mAh g^−1^ but also a long lifespan, maintaining 100% capacity after 20,000 cycles. More impressively, 75% of its specific capacity still remains even at a current density of 80 A g^−1^. Thanks to these excellent electrochemical performances, the fabricated AAZB achieves an energy density of 590 Wh kg^−1^ and a power density of 128.57 kW kg^−1^ (based on the mass loading of the active materials of the cathode).

## 2. Results and Discussion

**Figure 1:**
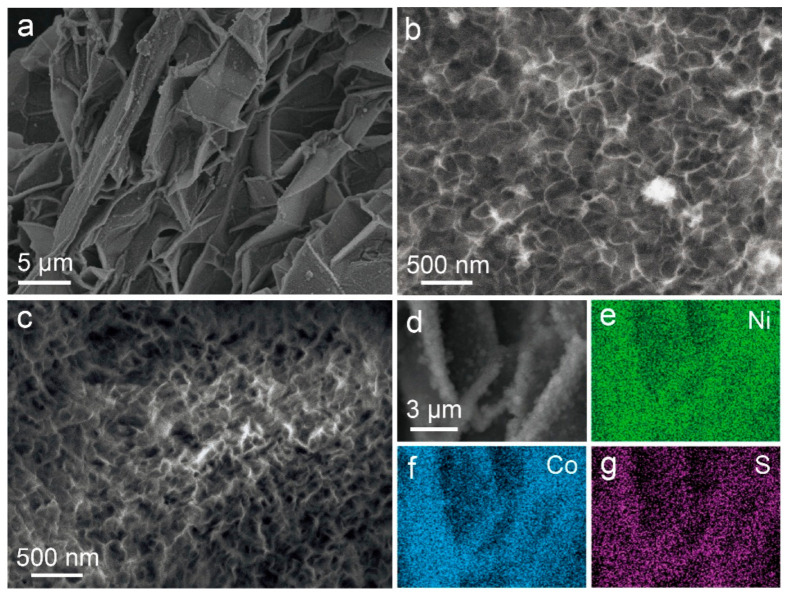
SEM images of (**a**,**b**) FLG@Ni_x_S_6_/Co_4_S_3_/Ni-Co(OH)_2_ and (**c**) FLG@Ni-Co(OH)_2_. (**d**–**g**) Elemental mapping of Ni, Co, and S in FLG@Ni_x_S_6_/Co_4_S_3_/Ni-Co(OH)_2_.

In this study, the cathode-exfoliated carbon electrode with few-layer graphene (FLG) on top, which serves as a current collector with a three-dimensional conductive network, has a porous structure. This porous structure provides an abundant surface area for the growth of active material, enabling the rapid transport of electrons and ions within the electrode. A series of electrode materials was synthesized through electrodeposition (Figure S1), with the concentration of thiourea in the electrolyte accurately adjusted. Among the prepared samples, the electrode fabricated using a deposition solution containing 30 mM thiourea exhibited excellent electrochemical energy storage performance, denoted here as FLG@Ni_x_S_6_/Co_4_S_3_/Ni-Co(OH)_2_ (see Figure S2), and was thus selected for further characterization. Figure 1a–c and Figure S3 show the scanning electron microscope (SEM) images of FLG@Ni_x_S_6_/Co_4_S_3_/Ni-Co(OH)_2_ and FLG@Ni-Co(OH)_2_. As shown, the nanosheets grow uniformly in situ on the FLG substrate. This hierarchical architecture provides abundant electrochemically active and well-interconnected sites, as well as multiscale ion/electron transport pathways. Furthermore, as shown in Figure S4, the related high-resolution transmission electron microscopy (HRTEM) image shows the lattice fringe spaces of 0.29, 0.30, 0.39, and 0.93 nm, which correspond to the (−151) plane of Ni_x_S_6_, (311) plane of Co_4_S_3_, (006) plane of Ni(OH)_2_·0.75H_2_O, and (001) plane of Co(OH)_2_, respectively. These features together promote rapid and directional charge transfer, thereby significantly enhancing the kinetics of electrochemical reactions. Moreover, the elemental distribution mapping of the FLG@Ni_x_S_6_/Co_4_S_3_/Ni-Co(OH)_2_ nanocomposite (Figure 1d–g) reveals a highly uniform spatial distribution of the S element throughout the material, providing direct experimental support for the homogeneous coexistence of constituent elements at length scales ranging from atomic to several nanometers.

**Figure 2:**
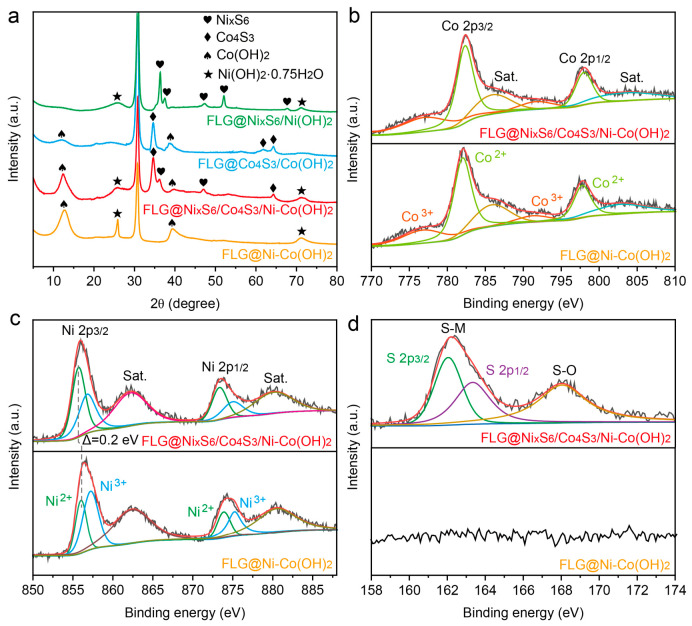
(**a**) XRD patterns of FLG@Ni_x_S_6_/Co_4_S_3_/Ni-Co(OH)_2_, FLG@Ni-Co(OH)_2_, FLG@Ni_x_S_6_/Ni(OH)_2_, and FLG@Co_4_S_3_/Co(OH)_2_. (**b**) Co 2p, (**c**) Ni 2p, and (**d**) S 2p of FLG@Ni_x_S_6_/Co_4_S_3_/Ni-Co(OH)_2_ and FLG@Ni-Co(OH)_2_.

The composition and chemical valences of the as-prepared electrodes were investigated via X-ray diffraction (XRD) and X-ray photoelectron spectroscopy (XPS) analysis. As shown in Figure 2a, the characteristic diffraction peaks of FLG@Ni_x_S_6_/Co_4_S_3_/Ni-Co(OH)_2_ are well-matched with those of Ni(OH)_2_·0.75H_2_O (JCPDS No. 38-0715) and Co(OH)_2_ (JCPDS No. 51-1731), which are also detected in the pristine FLG@Ni-Co(OH)_2_ sample. Moreover, distinct diffraction signals corresponding to Ni_x_S_6_ (JCPDS No. 51-0718) and Co_4_S_3_ (JCPDS No. 02-1338) can be clearly identified, confirming the successful synthesis of composite materials with in situ introduced sulfide components. The XPS survey spectrum in Figure S5 validates the coexistence of Ni, Co, O, and S elements in FLG@Ni_x_S_6_/Co_4_S_3_/Ni-Co(OH)_2_, which is consistent with the elemental mapping results. The XPS spectra were further analyzed to clarify the valence states of Ni and Co. The Co 2p spectrum can be deconvoluted into six peaks, consisting of two sets of spin–orbit splitting peaks (Co 2p_3_/_2_, Co 2p_1_/_2_) and two satellite peaks. Specifically, the binding energies at 785.8 eV (Co 2p_3_/_2_) and 802.7 eV (Co 2p_1_/_2_) are related to Co^2+^, while those at 776.7 eV and 791.3 eV are ascribed to Co^3+^ (Figure 2b) [2,26,27]. Similarly, the Ni 2p spectrum is decomposed into six peaks including main spin–orbit and satellite features (Figure 2c). The peaks located at 856.0 eV (Ni 2p_3_/_2_) and 873.8 eV (Ni 2p_1_/_2_) are related to Ni^2+^, and the peaks at 857.2 eV and 875.2 eV are assigned to Ni^3+^ [1,13,16,28]. Notably, the obvious binding energy shift of Ni 2p in the composite indicates the occurrence of interfacial charge redistribution. Such a strong electronic coupling effect within the composite structure is beneficial for optimizing the reaction kinetics and ultimately enhancing the electrochemical performance of the electrode [25,29,30]. Additionally, in the S 2p spectra (Figure 2d), the FLG@Ni_x_S_6_/Co_4_S_3_/Ni-Co(OH)_2_ exhibits two peaks at S 2p_3/2_ (162.1 eV) and S 2p_1/2_ (163.33 eV), which indicate the presence of metal–sulfur bonds. Moreover, the peak at 168.1 eV corresponds to oxidized sulfur species as a result of surface oxidation [24,25].

**Figure 3:**
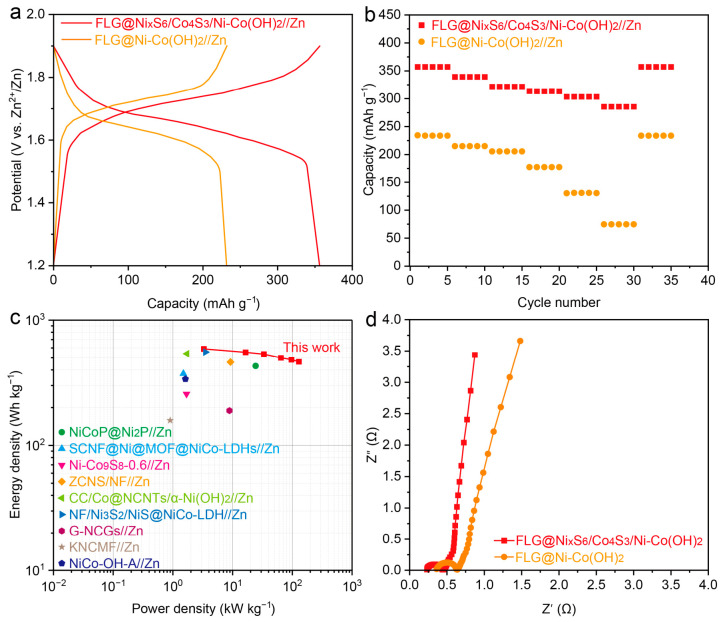
(**a**) GCD curves of FLG@Ni_x_S_6_/Co_4_S_3_/Ni-Co(OH)_2_//Zn and FLG@Ni-Co(OH)_2_//Zn. (**b**) Rate capability of FLG@Ni_x_S_6_/Co_4_S_3_/Ni-Co(OH)_2_//Zn and FLG@Ni-Co(OH)_2_//Zn at various densities. (**c**) Ragone plots of FLG@Ni_x_S_6_/Co_4_S_3_/Ni-Co(OH)_2_//Zn in comparison with previously reported Ni-Zn batteries. (**d**) EIS spectra of FLG@Ni_x_S_6_/Co_4_S_3_/Ni-Co(OH)_2_ and FLG@Ni-Co(OH)_2_.

The electrochemical energy storage capabilities of the as-obtained electrodes, when employed as the cathode for AAZBs, were investigated by using Zn foil as the anode with an electrolyte composed of 6 M KOH and 0.2 M Zn(CH_3_COO)_2_. As shown in Figure 3a, the galvanostatic charge–discharge (GCD) profiles of FLG@Ni_x_S_6_/Co_4_S_3_/Ni-Co(OH)_2_//Zn and FLG@Ni-Co(OH)_2_//Zn were compared at 2A g^−1^. The former delivers a specific capacity of 358 mAh·g^−1^, which substantially exceeded the 234 mAh·g^−1^ achieved by the latter (based on the mass loading of the active materials of the cathode). Rate capability (Figure 3b) evaluation further reveals that FLG@Ni_x_S_6_/Co_4_S_3_/Ni-Co(OH)_2_//Zn retained specific capacities of 358, 339, 321, 313, 303, and 287 mAh·g^−1^ at current densities of 2, 10, 20, 40, 60, and 80 A·g^−1^, respectively. It consistently outperforms its FLG@Ni-Co(OH)_2_//Zn counterpart across all tested rates, confirming its exceptional high current rate tolerance. The capacity of FLG@Ni_x_S_6_/Co_4_S_3_/Ni-Co(OH)_2_//Zn also exceeds those of other recently reported alkaline zinc batteries (Table S1), such as SCNF@Ni@MOF@NiCo-LDHs//Zn (342 mAh g^−1^ at 1.5 A g^−1^) [31], Ni_3_S_2_/Co_3_S_4_-Sv//Zn (220.6 mAh g^−1^ at 1 A g^−1^) [32], Ni-Co_9_S_8_-0.6//Zn (152 mAh g^−1^ at 1 A g^−1^) [27], CC/Co@NCNTs/α-Ni(OH)_2_//Zn (316 mAh g^−1^ at 1 A g^−1^) [26], NF/Ni_3_S_2_/NiS@NiCo-LDH//Zn (317.9 mAh g^−1^ at 2 A g^−1^) [25], G-NCGs//Zn (113.8 mAh g^−1^ at 0.5 A g^−1^) [22], Od-CNO@Ni NTs//Zn (334.9 mAh g^−1^ at 3 A g^−1^) [19], and NiCo-OH-A//Zn (208.7 mAh g^−1^ at 1 A g^−1^) [3]. In addition, FLG@Ni_x_S_6_/Co_4_S_3_/Ni-Co(OH)_2_//Zn attained an energy density of 590 Wh kg^−1^ at a power density of 3.295 kW kg^−1^ and a power density of 128.572 kW kg^−1^ at an energy density of 465 Wh kg^−1^ (Figure 3c). This performance is much superior to that of other reported alkaline zinc batteries, such as NiCoP@Ni_2_P//Zn (429.5 Wh kg^−1^ at 24.3 kW kg^−1^) [33], SCNF@Ni@MOF@NiCo-LDHs//Zn (373.2 Wh kg^−1^ at 1.5 kW kg^−1^) [34], Ni-Co_9_S_8_-0.6//Zn (256.5 Wh kg^−1^ at 1.69 kW kg^−1^) [27], ZCNS/NF//Zn (462 Wh kg^−1^ at 9.2 kW kg^−1^) [35], CC/Co@NCNTs/α-Ni(OH)_2_//Zn (540.4 Wh kg^−1^ at 1.72 kW kg^−1^) [26], NF/Ni_3_S_2_/NiS@NiCo-LDH//Zn (556.3 Wh kg^−1^ at 3.5 kW kg^−1^) [25], G-NCGs//Zn (189.25 Wh kg^−1^ at 8.84 kW kg^−1^) [22], KNCMF//Zn (158.2 Wh kg^−1^ at 0.9 kW kg^−1^) [23], and NiCo-OH-A//Zn (338 Wh kg^−1^ at 1.62 kW kg^−1^) [3]. Moreover, the superior electrochemical performance of the FLG@Ni_x_S_6_/Co_4_S_3_/Ni-Co(OH)_2_ was verified by the electrochemical impedance spectroscopy (EIS) data presented in Figure 3d. The FLG@Ni_x_S_6_/Co_4_S_3_/Ni-Co(OH)_2_ exhibits a small equivalent series resistance (ESR) value of 0.23 Ω in the high frequency region, which is smaller than that of the FLG@Ni-Co(OH)_2_ (0.36 Ω). This small ESR value implies an insignificant change in electron conductivity. In the high-frequency region, a smaller semicircle is observed for FLG@Ni-Co(OH)_2_, indicating faster charge transfer kinetics. Moreover, in the low-frequency region, the slope of FLG@Ni_x_S_6_/Co_4_S_3_/Ni-Co(OH)_2_ is steeper, demonstrating superior charge storage. Collectively, these EIS characteristics confirm the enhanced ionic diffusion kinetics and improved reversibility of redox reactions of FLG@Ni_x_S_6_/Co_4_S_3_/Ni-Co(OH)_2_.

**Figure 4:**
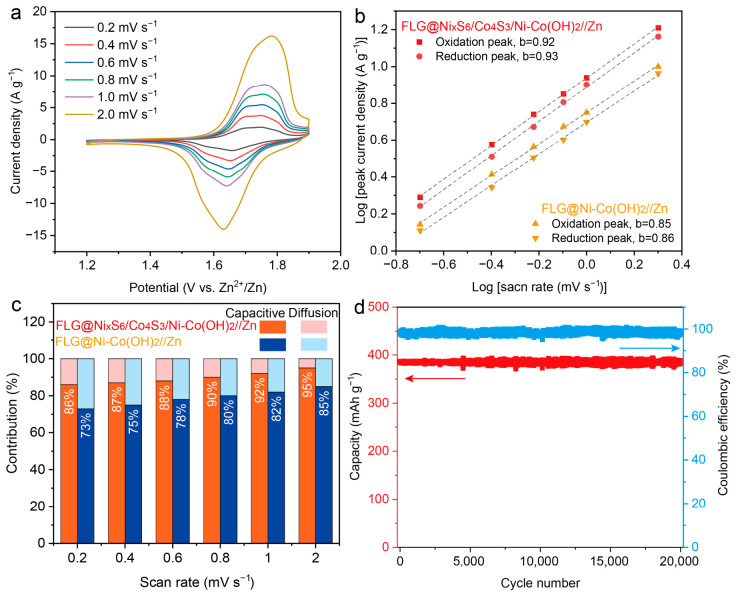
(**a**) CV profiles at various scan rates, (**b**) the curve of log (peak current density) vs log (scan rate), and (**c**) diffusion and capacitive contribution to the total current at different scan rates for FLG@Ni_x_S_6_/Co_4_S_3_/Ni-Co(OH)_2_//Zn and FLG@Ni-Co(OH)_2_//Zn. (**d**) The long-term cycling stability of FLG@Ni_x_S_6_/Co_4_S_3_/Ni-Co(OH)_2_//Zn.

The reaction kinetics of the battery were further investigated via cyclic voltammetry (CV) at scan rates spanning from 0.2 to 2.0 mV s^−1^ (Figure 4a and Figure S6). To elucidate the electrochemical kinetic mechanism of the battery system, the CV results were fitted in accordance with the power–law relationship *i* = a*v*^b^ between peak current (*i*) and scan rate (*v*), where a and b are adjustable fitting constants. Generally, the b value approaching 0.5 indicates a diffusion-controlled process, whereas the b value close to 1.0 signifies a surface capacitive-dominated behavior. For the FLG@Ni_x_S_6_/Co_4_S_3_/Ni-Co(OH)_2_//Zn battery, the b values calculated from the cathodic and anodic redox peaks are 0.92 and 0.93, respectively, indicating that its electrochemical redox kinetics are primarily governed by surface capacitive behavior. These values are better than those of previously reported AAZBs, demonstrating their more rapid reaction kinetics [26,31,32,35]. Moreover, the capacitive contribution (*k*_1_*v*) and diffusion-controlled contribution (*k*_2_*v*^1/2^) can be quantitatively separated based on the equation *i* = *k*_1_*v* + *k*_2_*v*^1/2^, where *i* represents the current response at a given potential. The quantitative results show that the capacitive contribution ratios range from 86% to 95% at scan rates from 0.2 to 2.0 mV·s^−1^, respectively, showing a continuous increase as the scan rate rises. Moreover, cycle stability is a crucial performance metric for evaluating AAZBs. As shown in Figure 4d, FLG@Ni_x_S_6_/Co_4_S_3_/Ni-Co(OH)_2_//Zn demonstrates outstanding long-term cycling stability. After 20,000 cycles at a current density of 2 A g^−1^, the discharge capacity remains almost unchanged, and the Coulombic efficiency is maintained at approximately 100% throughout the whole test period, which shows superior electrochemical reversibility. Furthermore, the electrode structure exhibits remarkable integrity and cycling stability (Figure S7), which provides strong support for maintaining a high capacity retention even after 20,000 cycles.

## 3. Materials and Methods

### 3.1. Materials

Graphite foil (GF) was procured from SGL Carbon GmbH (Meitingen, Germany). LiClO_4_ was obtained from Shanghai Aladdin Chemicals (Shanghai, China). All other chemical reagents were sourced from Sinopharm Chemical Reagent Co., Ltd. (Shanghai, China).

### 3.2. Cathode-Exfoliated Carbon Electrode with Few-Layer Graphene (FLG)

Electrochemical exfoliation was carried out in a two-electrode system. A GF with dimensions of 0.8 cm × 0.8 cm was used as the working electrode, while a Pt sheet acted as the counter electrode. The electrolyte was composed of propylene carbonate with a concentration of 150 mg mL^−1^ LiClO_4_. A constant cathodic potential of −10 V was applied under ambient conditions for 25 s to trigger graphite exfoliation and produce expanded FLG. After exfoliation, the FLG product was successively rinsed with anhydrous ethanol and deionized water. Residual electrolyte and electrochemical by-products were then removed by immersing the product in a 1 M HCl solution at room temperature for 6 h. Subsequently, the sample was repeatedly washed with deionized water until the pH was confirmed to be neutral, and finally freeze-dried to obtain the FLG.

### 3.3. Electrochemical Codeposition of FLG@Ni_x_S_6_/Co_4_S_3_/Ni-Co(OH)_2_

Electrochemical codeposition was carried out in a conventional three-electrode system with FLG as the working electrode and GF as the counter electrode. Cyclic voltammetry (CV) was performed at a scan rate of 50 mV s^−1^ within a potential window of −1.3 to 0.2 V (vs. SCE). The electrolyte consisted of 40 mM Ni(NO_3_)_2_ and 20 mM Co(NO_3_)_2_, supplemented with varying concentrations of thiourea, namely, 0, 10, 20, 30, and 40 mM, to regulate sulfur incorporation during electrodeposition. The resultant composite electrodes were systematically named as follows: FLG@Ni-Co(OH)_2_, FLG@Ni_x_S_6_/Co_4_S_3_/Ni-Co(OH)_2_-10 mM, FLG@Ni_x_S_6_/Co_4_S_3_/Ni-Co(OH)_2_-20 mM, FLG@Ni_x_S_6_/Co_4_S_3_/Ni-Co(OH)_2_-30 mM, and FLG@Ni_x_S_6_/Co_4_S_3_/Ni-Co(OH)_2_-40 mM. Additionally, two extra electrodes, FLG@Ni_x_S_6_/Ni(OH)_2_ and FLG@Co_4_S_3_/Co(OH)_2_, were prepared under the same electrochemical conditions, but with a thiourea concentration of 30 mM and electrolytes containing 60 mM Ni(NO_3_)_2_ or 60 mM Co(NO_3_)_2_, respectively.

### 3.4. Material Characterization

The morphologies of the samples were examined using a scanning electron microscope (SEM, SU 8010, HITACHI, Tokyo, Japan) at an accelerating voltage ranging from 5 to 10 kV. The microstructures were characterized using high-resolution transmission electron microscopy (HRTEM, JEM-2100F, JEOL, Tokyo, Japan) at an acceleration voltage of 200 kV. X-ray photoelectron spectroscopy (XPS) measurements were carried out on an ESCA-LAB 250Xi spectrometer (Thermo Scientific, Waltham, MA, USA) with monochromatic Al Kα radiation (λ = 8.34 Å) serving as the excitation source. All XPS spectra were calibrated by referring to the C 1s peak at 284.8 eV to correct the charging effect. X-ray diffraction (XRD) patterns were acquired on a Bruker D8 Advance X’Pert Pro diffractometer with Cu Kα radiation, operating at a scanning rate of 5° min^−1^ and a 2θ range from 5° to 80°.

### 3.5. Electrochemical Tests

The electrochemical performance tests of AAZBs were carried out using a two-electrode system. The electrolyte was composed of an aqueous solution containing 6 M KOH and 0.2 M Zn(CH_3_COO)_2_. The as-prepared electrodes were utilized as the cathode, and a Zn foil was used as the anode. Electrochemical impedance spectroscopy (EIS) analysis of FLG@Ni-Co(OH)_2_ and FLG@Ni_x_S_6_/Co_4_S_3_/Ni-Co(OH)_2_ was carried out in a three-electrode system. A plate was employed as the counter electrode, and a Hg/HgO electrode was used as the reference electrode. The measurements were carried out at the open-circuit potential within a frequency range from 0.01 Hz to 100 kHz, with a perturbation of 10 mV. All electrochemical tests were performed using a CHI 660E electrochemical workstation or a LAND CT2001A battery testing system.

### 3.6. Calculations

The specific capacity (*C_m_*, mAh g^−1^), energy density (*E*, Wh kg^−1^) and power density (*P*, kW kg^−1^) were obtained by the following formulas:

(1) $$C_{m}=\frac{I\Delta t}{m}$$

(2) $$E=C_{m}\times \Delta V$$

(3) $$P=\frac{3.6\times E}{\Delta t}$$

where *I* represents the discharge current (*A*), Δ*t* represents the discharge time (*h*), and *m* represents the mass (*g*) of the active material.

## 4. Conclusions

In summary, we have successfully synthesized a high-performance cathode material, namely FLG@Ni_x_S_6_/Co_4_S_3_/Ni-Co(OH)_2_ nanosheets, which form a composite through a facile electrochemical approach. The strong composite formed by the constituent active phases significantly enhances both electronic and ionic transport kinetics. Meanwhile, it creates a high density of accessible electrochemical reaction sites. Consequently, the FLG@Ni_x_S_6_/Co_4_S_3_/Ni-Co(OH)_2_//Zn delivers a high discharge specific capacity of 385 mAh g^−1^ at 2 A g^−1^, along with excellent rate capability, notably outperforming the benchmark FLG@Ni-Co(OH)_2_//Zn counterpart. Moreover, the device attains a maximum energy density of 590 Wh kg^−1^ and a maximum power density of 128.572 kW kg^−1^. Notably, it retains 100% of its capacity after 20,000 consecutive cycles at 2 A g^−1^, indicating remarkable long-term cycling stability. Looking forward, such multiphase collaborative design strategies can be applied to other electrochemical energy storage systems (e.g., solid-state batteries, sodium/potassium-ion batteries, etc.) to systematically evaluate their universality and adaptability under wide temperature ranges, high rates, and different electrolyte environments. Meanwhile, it is of urgent necessity to conduct process feasibility studies for large-scale production, including optimizing precursor usage, controlling electrode coating uniformity, verifying batch consistency, and performing cost–benefit analysis, to comprehensively assess the engineering potential and industrialization prospects of this preparation route.

## Data Availability

The data presented in this study are available upon request from the corresponding author. The data are not publicly available due to commercial reasons.

## Supplementary Materials

The following supporting information can be downloaded at: https://www.mdpi.com/article/10.3390/molecules31111829/s1, Figure S1: Synthesis mechanism of FLG@Ni_x_S_6_/Co_4_S_3_/Ni-Co(OH)_2_; Figure S2: (a–e) GCD curves of FLG@Ni-Co(OH)_2_ and FLG@Ni_x_S_6_/Co_4_S_3_/Ni-Co(OH)_2_ (with 10 mM, 20 mM, 30 mM and 40 mM thiourea concentrations) at different current densities. (f) Comparison of specific capacities of the samples; Figure S3: Cross-sectional SEM image of FLG@Ni_x_S_6_/Co_4_S_3_/Ni-Co(OH)_2_; Figure S4: HRTEM image of FLG@Ni_x_S_6_/Co_4_S_3_/Ni-Co(OH)_2_; Figure S5: XPS survey spectrum of FLG@Ni_x_S_6_/Co_4_S_3_/Ni-Co(OH)_2_ and FLG@Ni-Co(OH)_2_; Figure S6: CV profiles of FLG@Ni-Co(OH)_2_//Zn at various scan rates; Figure S7: SEM image of FLG@Ni_x_S_6_/Co_4_S_3_/Ni-Co(OH)_2_ after 20,000 cycles; and Table S1: Comparison of the electrochemical performance of alkaline zinc batteries.
