# Supplementary material for: A Multiphase Composite for High-Performance Alkaline Zinc Batteries
Source: Molecules. 2026 May 26;31(11):1829. doi: 10.3390/molecules31111829 (PMC13258467; doi:10.3390/molecules31111829)
Supplement: Supplementary file 1 [file molecules-31-01829-s001.zip › molecules-4326525-supplementary.pdf]

A Multiphase Composite for High-Performance Alkaline Zinc Batteries

Zhen Sun[1], Junran Wang[1], Jietao Guan[1], Yaoda Mei[1], Wenyu Song[1], Haixu Wang[1], Weiwei Luo[1], and Xiang Cai [1,2,*]

[1] Liaoning Key Laboratory of Development and Utilization for Natural Products Active Molecules, School of Chemistry and Life Science, Anshan Normal University, Anshan 114005, China;

[2] School of Light Industry and Chemical Engineering, Dalian Polytechnic University, Dalian 116034, China;

* Correspondence: caixiang@dlpu.edu.cn

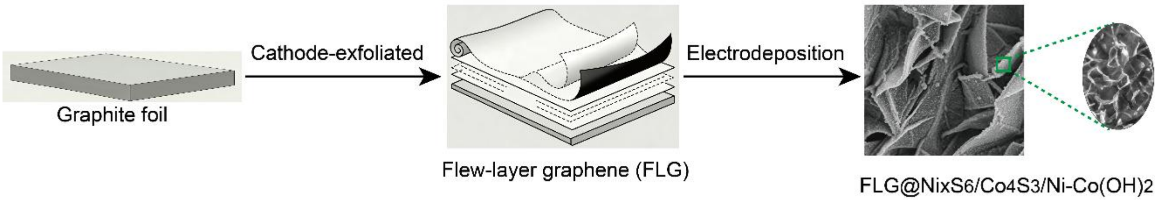

Figure S1. Synthesis mechanism of FLG@Ni_xS_6/Co_4S_3/Ni-Co$(OH)_2$.

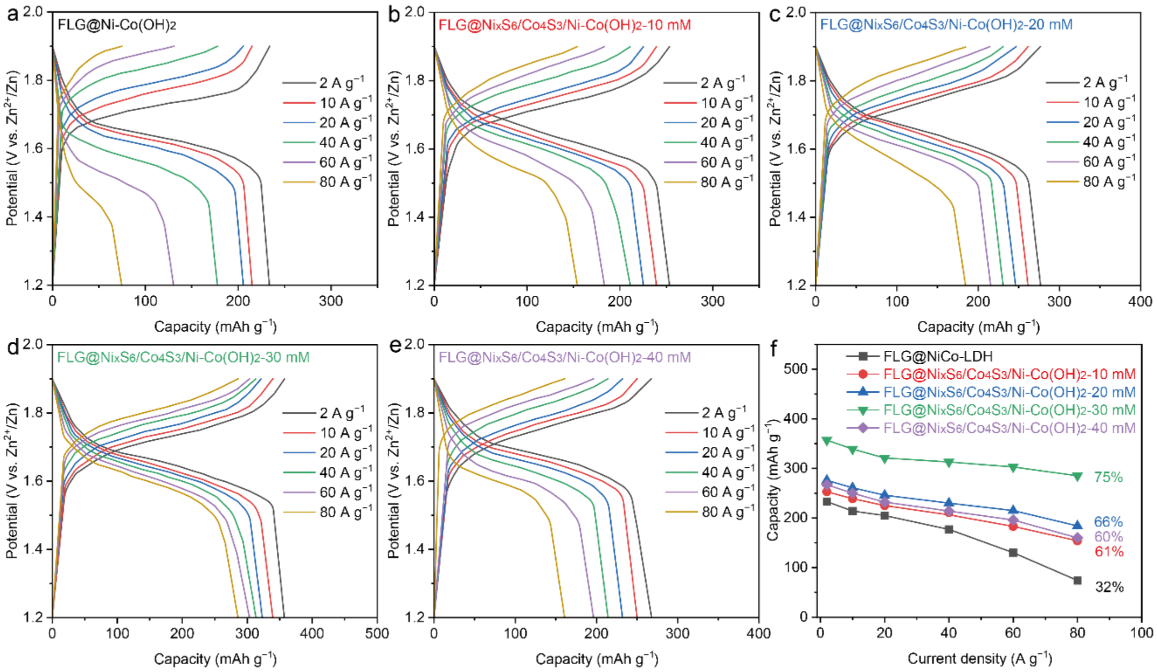

Figure S2. (a-e) GCD curves of FLG@Ni-Co$(OH)_2$ and FLG@Ni_xS_6/Co_4S_3/Ni-Co$(OH)_2$ (with 10 mM, 20 mM, 30 mM and 40 mM thiourea concentrations) at different current densities. (f) Comparison of specific capacities of the samples.

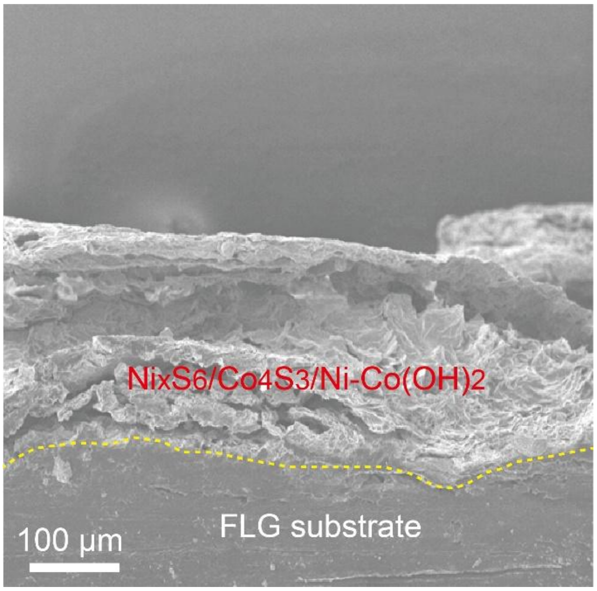

Figure S3. Cross-sectional SEM image of FLG@Ni_xS_6/Co_4S_3/Ni-$Co(OH)_2$.

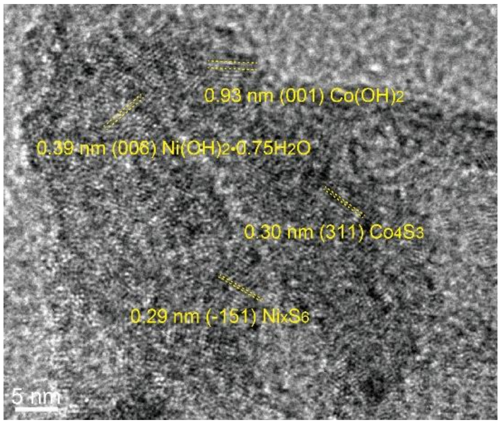

Figure S4. HRTEM image of FLG@Ni_xS_6/Co_4S_3/Ni-$Co(OH)_2$.

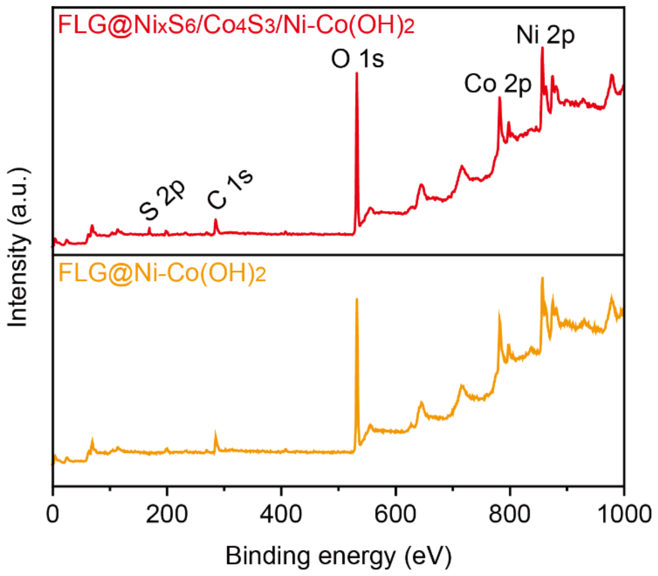

Figure S5. XPS survey spectrum of FLG@Ni_xS_6/Co_4S_3/Ni-$Co(OH)_2$ and FLG@Ni-$Co(OH)_2$.

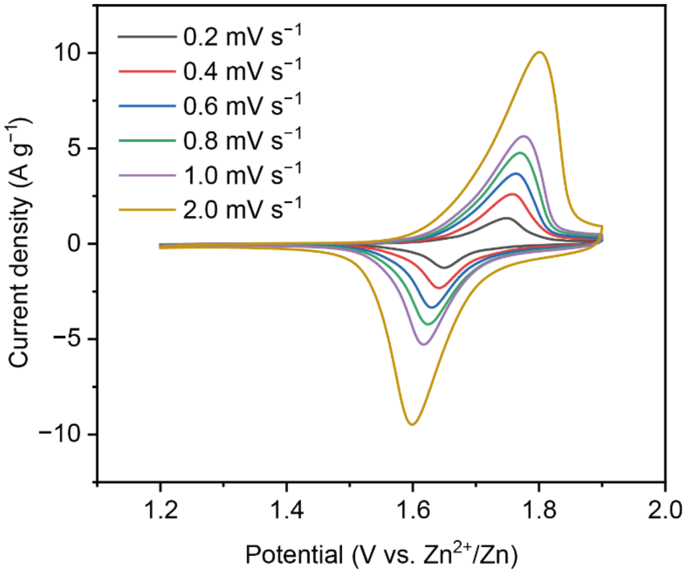

Figure S6. CV profiles of FLG@Ni-$Co(OH)_2$//Zn at various scan rates.

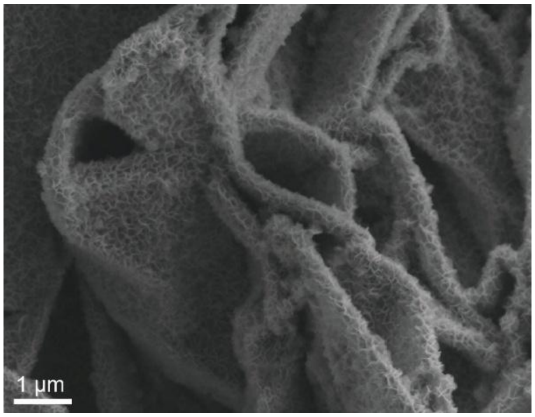

Figure S7. SEM image of FLG@Ni_xS_6/Co_4S_3/Ni-Co$(OH)_2$ after 20,000 cycles.

Table S1. Comparison of the electrochemical performance of alkaline zinc batteries.

Cathode//Zn	Capacity (mAh g^{-1})	Rate performance	E_{max} (Wh kg^{-1})	P_{max} (kW kg^{-1})	Cycling life
This work	385 (2 A g^{-1})	75% (80 A g^{-1})	590	128.57	100% (20,000)
NiCo-OH-A//Zn[1]	208.7 (1 A g^{-1})	45.5% (20 A g^{-1})	338	29.89	76.5% (2,000)
Od-CNO@Ni NTs//Zn[2]	334.9 (3 A g^{-1})	48% (60 A g^{-1})	547.5	92.9	64% (10,000)
G-NCGs//Zn[3]	113.8 (0.5 A g^{-1})	38% (5 A g^{-1})	189.25	8.84	50% (2,000)
NF/Ni_3S_2/NiS@NiCo-LDH//Zn[4]	317.9 (2 A g^{-1})	32% (15 A g^{-1})	556.3	26.3	116.7% (5,000)
CC/Co@NCNTs/α-Ni$(OH)_2$//Zn[5]	316 (1 A g^{-1})	75% (10 A g^{-1})	540.4	——	84% (2,000)
Ni-Co_9S_8-0.6//Zn[6]	152 (1 A g^{-1})	64% (10 A g^{-1})	256.5	16.56	92% (3,000)
Ni_3S_2/Co_3S_4-Sv//Zn[7]	220.6 (1 A g^{-1})	86.3% (5 A g^{-1})	59.5	4	104% (2,000)
SCNF@Ni@MOF@NiCo-LDHs//Zn[8]	342 (1.5 A g^{-1})	65% (38.2 A g^{-1})	572.5	62.81	55% (5,000)
NiCoP@Ni_2P//Zn[9]	358.3 (1 A g^{-1})	63% (20 A g^{-1})	596.3	24.3	89% (10,000)
SCNF@Ni@MOF@NiCo-LDHs//Zn[10]	215 (5 A g^{-1})	83% (10 A g^{-1})	373.2	——	82% (100)
ZCNS/NF//Zn[11]	——	——	462	——	82% (2,000)
KNCMF//Zn[12]	——	——	158.2	27.1	132% (5,000)

References

1. Diao, Y.; Cao, H.; Zhang, C.; Zhang, C.; Chen, H. C. In Situ Construction of Hierarchical Nickel-Cobalt Hydroxides Derived from Metal-Organic Frameworks for High-Performance Nickel-Zinc Batteries. *ChemSusChem* **2025**, *18*, e202401612.
2. Yao, J.; Wan, H.; Chen, C.; Ji, J.; Wang, N.; Zheng, Z.; Duan, J.; Wang, X.; Ma, G.; Tao, L.; et al. Oxygen-Defect Enhanced Anion Adsorption Energy Toward Super-Rate and Durable Cathode for Ni-Zn Batteries. *Nano-Micro Lett.* **2021**, *13*, 167.
3. Zhang, X.; He, J.; Zhou, L.; Zhang, H.; Wang, Q.; Huang, B.; Lu, X.; Tong, Y.; Wang, C. Ni (II) Coordination Supramolecular Grids for Aqueous Nickel-Zinc Battery Cathodes. *Adv. Funct. Mater.* **2021**, *31*, 2100443.
4. Zhou, K.; Wang, S.; Zhong, G.; Chen, J.; Bao, Y.; Niu, L. Hierarchical Heterostructure Engineering of Layered Double Hydroxides on Nickel Sulfides Heteronanowire Arrays as Efficient Cathode for Alkaline Aqueous Zinc Batteries. *Small* **2022**, *18*, 2202799.
5. Zhu, L.; Fei, B.; Xie, Y.; Cai, D.; Chen, Q.; Zhan, H. Engineering Hierarchical Co@N-Doped Carbon Nanotubes/α-$Ni(OH)_2$ Heterostructures on Carbon Cloth Enabling High-Performance Aqueous Nickel-Zinc Batteries. *ACS Appl. Mater. Interfaces* **2021**, *13* (19), 22304-22313.
6. Zhang, D.; Zhang, J.; Li, J.; Li, C.; Li, Y.; Liu, Y.; Zhang, R. Facile Synthesis of Mesoporous $Ni_xCo_{9-x}S_8$ Hollow Spheres for High-Performance Supercapacitors and Aqueous Ni/Co-Zn Batteries. *RSC Adv.* **2022**, *12* (32), 20447-20453.
7. Liang, M.; Li, Z.; Kang, Y.; Zhao, X.; Zhang, X.; Zhang, H.; Wang, H.; Miao, Z.; Fu, C. Ni_3S_2/Co_3S_4 with Controlled Surface Electron Arrangement for High-Performance Aqueous Energy Storage. *J. Mater. Chem. A* **2024**, *12* (8), 4623-4634.
8. Xia, Z.; Pan, J.; Chen, H.; Deng, N.; Yang, C.; Liu, X.; Liu, Y.; Wu, L. Flexible One-Dimensional Yarn-Like Ni-Zn Battery: Micron-Nano Hierarchical-Structure Array, High Energy Density and Excellent Capacity Retention. *Chem. Eng. J.* **2023**, *456*, 141048.
9. Li, C.; Zhao, S.; Zhang, X.; Qu, G.; Li, X.; Li, N.; Wang, T.; Leng, J.; Wang, C.; Xu, X. Advanced Aqueous Zinc Battery with Excellent Rate and Low-Temperature Adaptation Enabled by Bimetallic Phosphide with Hetero-Interface. *Chem. Eng. J.* **2022**, *450*, 137998.
10. Bao, Y.; Zhang, W.; Yun, T.; Dai, J.; Li, G.; Mao, W.; Guan, M.; Zhuang, Y. The Application of Transition Metal Sulfide Ni_3S_4/CNFs in Rechargeable Ni-Zn Batteries. *New J. Chem.* **2021**, *45* (47), 22491-22496.
11. Zhou, Y.; Tong, X.; Pang, N.; Deng, Y.; Yan, C.; Wu, D.; Xu, S.; Xiong, D.; Wang, L.; Chu, P. K. Ni_3S_2 Nanocomposite Structures Doped with Zn and Co as Long-Lifetime, High-Energy-Density, and Binder-Free Cathodes in Flexible Aqueous Nickel-Zinc Batteries. *ACS Appl. Mater. Interfaces.* **2021**, *13* (29), 34292-34300.
12. Yu, W.; Ding, R.; Jia, Z.; Li, Y.; Wang, A.; Liu, M.; Yang, F.; Sun, X.; Liu, E. Pseudocapacitive Co-Free Trimetallic Ni-Zn-Mn Perovskite Fluorides Enable Fast-Rechargeable Zn-Based Aqueous Batteries. *Adv. Funct. Mater.* **2022**, *32*, 2112469.
